# A triangulated perspective for understanding CAM use in Lebanon: a qualitative study

**DOI:** 10.1186/s12906-022-03685-z

**Published:** 2022-08-02

**Authors:** Zeinab Jaafar, Jennifer Ayoub, Rena Hamadeh, Samar Baydoun, Gladys Honein-AbouHaidar, Jinan Banna, Mohamad Alameddine, Farah Naja

**Affiliations:** 1 Health Promotion Department, CAPHRI, Maastricht University, Maastricht, The Netherlands; 2 Nutrition and Food Sciences Department, American University of Beirut, Beirut, Lebanon; 3 Hariri School of Medicine, American University of Beirut, Beirut, Lebanon; 4 Department of Human Nutrition, Food and Animal Sciences, College of Tropical Agriculture and Human Resources, University of Hawaii, Manoa, Honolulu, HI 96822 USA; 5 College of Health Sciences, University of Sharjah, Sharjah, United Arab Emirates; 6 Department of Clinical Nutrition and Dietetics, College of Health Sciences, Research Institute of Medical & Health Sciences (RIMHS), University of Sharjah, Sharjah, United Arab Emirates; 7 Department of Nutrition and Food Sciences, Faculty of Agricultural and Food Sciences, American University of Beirut, Beirut, Lebanon

**Keywords:** Complementary and alternative medicine, Qualitative, Lebanon, Middle east

## Abstract

**Background:**

Existing evidence marked a prevalent use of Complementary and Alternative Medicine (CAM) therapies in Lebanon that is concomitant with low rates of disclosure to health care providers and limited knowledge among the general public of safety and side effects of CAM use.

**Objectives:**

To examine the perspectives of Lebanese CAM users, CAM providers, and health care providers (HCPs) regarding their understanding of CAM and of the *Push* and *Pull* factors that drive its use.

**Methods:**

A qualitative research study was conducted using in-depth interviews, targeting Lebanese adults (CAM users; 18-65 years) (*n*=14), CAM providers such as yoga instructors, owners of CAM product outlets, herbalists, and religious figures (*n*=13); and HCPs including physicians, nurses, dietitians, and pharmacists (*n*=14). The topic guide covered, in addition to the understanding of CAM, the *Push* and *Pull* factors driving CAM use. The adults were recruited by convenient sampling, and CAM providers and HCPs using a purposive sampling approach. Interviews were audiotaped, transcribed, and translated into English. Analysis was performed using a qualitative thematic approach. Similarities and differences in the perceptions of the participants with regards to factors that influence CAM use were charted and contrasted, using a triangulated approach.

**Results:**

The three study groups exhibited a similar understanding of CAM, referring to non-conventional therapies used to prevent/treat diseases or to enhance wellbeing. CAM users and CAM providers identified “distrust in HCPs”, “lack of patient-centered care in CM”, and “limitations and side effects of CM” as important *Push* factors. All study groups highlighted the limited CAM knowledge of HCPs as a main reason for the lack of patient-centered care. All three groups also underscored the affordability and the social and cultural support for CAM as main enablers of its prevalent use. Unlike HCPs who were skeptical about the safety and effectiveness of CAM, CAM users and CAM providers indicated that most of CAM therapies are safe and efficient.

**Conclusions:**

The triangulation of perspectives (CAM users, CAM providers, and HCPs) in this study allowed a comprehensive appraisal of CAM use and its drivers. Improving the HCPs’ CAM-related knowledge, promoting patient-centered care and fostering an open dialogue between HCPs and CAM providers are among the recommendations of the study.

**Supplementary Information:**

The online version contains supplementary material available at 10.1186/s12906-022-03685-z.

## Background

Despite some variability in the definition of Complementary and Alternative Medicine (CAM), it is generally agreed that these therapies include non-conventional products and therapies that are used either with or instead of mainstream medicine. Such therapies could be used for the treatment or prevention of diseases [1]. According to the National Institutes of Health, CAM is defined as “a group of diverse medical and health care systems, practices, and products that are not generally considered part of conventional medicine” (CM) [2]. The World Health Organization (WHO) categorizes CAM into therapies that involve medications (herbal, animal parts, or mineral) and non-medication therapies (acupuncture, manual therapies, or spiritual therapies) [3]. The American National Center for CAM therapies further groups CAM therapies into four categories: - 1) mind-body systems; - 2) manipulative and body-based practices; - 3) energy medicine; and - 4) biologically based practices [4].

Worldwide, despite some geographical variations, the use of CAM therapies has been increasing substantially among patients as well as healthy individuals [5, 6]. In high-income countries, growing numbers of patients rely on CAM, with estimates reaching 70-80% using at least one form of CAM [7, 8]. Estimates from low- and middle-income countries (LMICs) are also high; for instance, available evidence indicated that up to 80% of the people in Africa and Asia use CAM/traditional medicine to help complement their health care needs [3]. The popularity of CAM use is partly due to historical, social, and cultural reasons [7]**.** Additional factors include the challenges of CM in providing a cure or solution for human diseases and the almost unavoidable side effects of certain mainstream treatments [7]. In LMICs, the increasing use of CAM also relates to the lack of access to essential CM, shortage and mal-distribution of health care providers (HCPs) and health care facilities, and the relatively affordable cost of CAM therapies as compared to CM [9]. Published literature revealed that the low cost of CAM is one of the main reasons for its use among Asian and African populations [7]. In Lebanon, a previous study showed that diabetic patients use CAM therapies for the treatment of diabetes due to its low cost as compared to modern medicine [10]. In many instances, the use of CAM seems to strengthen the perception of one’s personal control over the course of health and disease, and to positively influence the quality of life [11].

Despite the potential beneficial effect that CAM use may have on health and quality of life, such use must be considered in the context of the associated risks. CAM use may interfere with the success of mainstream/conventional treatments as a result of interactions, and CAM use may impede uptake or adherence to mainstream medicine. The WHO indicated that there are significant challenges regarding the effective and safe use of CAM around the world, namely the development and enforcement of policies, safety and quality, qualification of practitioners, ability to control and regulate CAM advertising and claims, lack of scientific evidence on CAM therapies, as well as CAM-related education and training of HCPs [12].

The Middle East and North Africa (MENA) region hosts one of the fastest growing markets of CAM products in the world [13]. In Lebanon, a small country of the region, the use of CAM for different health purposes have also been increasingly popular. A national study from 2015 revealed that about one third of Lebanese adults (29.87%) use CAM [14]. Based on previous studies, the most reported forms of CAM products used in Lebanon include herbs and herbal products, dietary supplements, special foods, and spiritual healing practices [15, 16]. Higher rates of CAM use have also been reported among chronic disease patients including diabetes (38%) [16], infertility (41%) [17], breast cancer (40%) [15], lung cancer (41%) [18], and HIV and AIDS conditions (46.6%) [19]. In addition to the common use of CAM, the aforementioned studies commonly highlighted the use of CAM as an alternative to conventional medicine, the lack of knowledge regarding the toxic effects of certain CAM therapies and CAM-drug interactions, and the low disclosure of CAM use to HCPs in Lebanon [14, 17, 19–22]. Unfortunately, the popularity of CAM products in Lebanon has not been coupled with educational and regulatory mechanisms to better integrate those products into mainstream medicine [14]. While CAM regulation presents a challenge to most countries globally, regulation is particularly challenging in a country that suffers from a weak public sector, a fragmented healthcare system, ineffective health policies and politics, and a lack of coordination between the responsible ministries and stakeholders, all jeopardizing the safety of public consumption of CAM and hindering the integration of CAM therapies into mainstream medicine [23]. This is especially worrisome in the midst of the worst economic crisis the country has ever witnessed, causing dramatic rises in out-of-pocket healthcare costs, outpacing inflation and salaries, with more than 50% of the population left uninsured from any type of health coverage, which could further contribute to increased dependence on CAM as cheaper and accessible alternatives to conventional medicine.

Although the aforementioned studies provided valuable insights regarding CAM use in Lebanon, the quantitative methodologies used were mostly based on cross sectional surveys, and may have not captured the subtle and complex processes involved in CAM use [24, 25]. Using an exploratory approach, qualitative methods allow for more openness and flexibility to better understand human behavior. Qualitative studies are applied when answers about the why and how of individual experiences are needed. Particularly in the context of CAM, qualitative methods provide a more profound understanding of “subjectivity and complexity within the human experience, making them a powerful tool for increasing our knowledge of important processes within CAM” [24, 25].

Whenever available, the qualitative and quantitative studies addressing CAM use focused mainly on either the CAM users, HCPs, the CAM providers, or CAM regulators [26–28]. These studies usually address the uptake of CAM from a single perspective. For instance, solely examining the HCPs’ perspectives could result in skepticism and apprehension with regards to using CAM [28]. On the other hand, studies focusing on CAM providers would advocate for the promotion of CAM and its integration with standard care [29]. As for those studies that examined the general and/or patient populations, the recommendations could fall across a wide spectrum depending on the context, the beliefs, and attitudes of their study populations. As such, given the complexity of CAM use and the many stakeholders involved in the uptake of CAM, it is sensible to examine the perspectives of these stakeholders together in order to identify and bridge the gaps in knowledge and communication, as well as explore possible opportunities to create a coherent and effective momentum that fosters a safe and effective use of CAM. The main objective of this study was to examine the perspectives of CAM users, CAM providers, and HCPs regarding their understanding of CAM and of the *Push* and *Pull* factors that drive its use. Such a population triangulation will allow an in-depth examination of CAM use within the Lebanese context from a holistic lens. Results of this study aim to inform recommendations and public health policies to promote the safety of CAM users. It also aimed at providing essential knowledge to integrate CAM into CM.

## Methods

### Study design and approach

This study used a descriptive qualitative research design which allows researchers to comprehensively investigate complicated issues among participants within their dynamic cultural contexts and provide a deeper understanding by focusing on values and opinions. Drawing on individual in-depth interviews, the use of CAM was explored through a triangulated approach using the perceptions of: (a) CAM users, (b) CAM providers; and (c) HCPs. The in-depth interviews reflected the participants’ understanding of CAM and the factors that influence its use. For the purpose of this study, CAM use was defined as the use of any product or practice that is not considered to be part of conventional medicine for the purpose of preventing or managing any health condition or lessening any symptom in the 12 months prior to the interview.

### Sampling and recruitment

The recruitment of CAM users was carried out using convenience sampling while a purposive sampling frame was used to identify and recruit CAM providers and HCPs. To recruit CAM users, flyers were posted around the university and shared on the American University of Beirut’s Faculty of Agricultural and Food Sciences Facebook page. As for CAM providers, they were recruited from the catchment areas of the American University of Beirut Medical Center (AUBMC) as well as from the Makassed General Hospital (MGH) using purposive sampling. Flyers were posted at wellness centers and CAM outlets in the catchment areas of AUBMC and MGH. Furthermore, Dar Al Fatwa and Maronite Patriarchate were contacted to nominate two cheikhs and two priests, respectively. As for healthcare workers, they were invited via a departmental meeting that took place at each hospital (AUBMC and MGH). Pharmacists were recruited using flyers posted within the catchment areas of AUBMC and MGH.

**Fig. 1:**
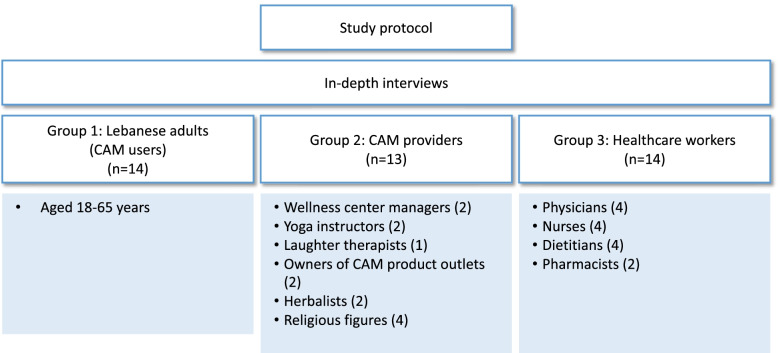
Study protocol and sampling of participants

To be eligible to participate in group 1 (G1) (CAM users), subjects were required to be Lebanese adults (aged 18 years or above) and conversant in Arabic language. CAM users were identified based on whether they have used any CAM modality (defined as any product or practice that is not considered to be part of conventional medicine) for the purpose of preventing or managing any health condition or lessening any symptom in the 12 months prior to the interview. For group 2 (G2), subjects were selected if they were providing any form of CAM. As such, providers of common CAM modalities were identified and invited to participate and included wellness center managers, yoga instructors, laughter therapists, owners of CAM product outlets, herbalists, and religious figures. For the third and last group (G3) (HCPs), various members of the health care team who provided services to patients were contacted and invited to take part in the study (physicians, nurses, dietitians, and pharmacists). The HCPs were selected from the two largest private and governmental medical centers in Beirut (American University of Beirut Medical Center and Makassed General Hospital). For the three groups, efforts were exerted to include both male and female participants, as well as participants who belong and/or serve different socio-economic levels. All subjects were asked to complete a short sociodemographic questionnaire to collect data on their age, country of birth, highest education level, employment status, residential location, and economic status. Recruitment in the three groups of sample populations continued until saturation of themes, whereby the addition of new members added little to the existing data collected; which resulted in 14 CAM users, 13 CAM providers, and 14 HCPs. Figure 1 summarizes the study protocol and sampling of participants.

### Interview guide

The interviews were semi-structured in nature, with pre-determined open-ended questions and prompts to explore the participants’ perceptions and experiences of CAM therapies. The topic guide and its questions were formulated following a thorough review of relevant literature with specific focus on gaps identified in previous quantitative studies conducted in Lebanon [30]. The topic guide, hence, addressed two main broad areas in relation to CAM: general understanding of CAM and the factors that affect its use in Lebanon. For the CAM understanding section, participants were asked to provide a definition of CAM and its types. In addition, in this section, for CAM providers and HCPs, a question regarding any training/education in relation to CAM was included. For the factors that affect CAM use section, the questions addressed two main areas: The *Push* factors (those that push people away from CM) or *Pull* factors (those that pull people towards CAM use). The *Push* and *Pull* factors approach adopted in the topic guide was based on the health belief model, whereby health beliefs were postulated as key determinants of health behavior. In fact, the health belief model, and specifically the *Push* and *Pull* model, have been previously used to investigate and better understand the motives and deterrents behind CAM use [31]. A copy of the interview guide is added as Additional file 1.

### Data collection

The in-depth interviews were carried out by three research assistants, between June and August 2019 in the city of Beirut. They were trained on how to conduct an interview including how to maintain a neutral and non-judgmental attitude while keeping the interviewees engaged and interested in the topic. JA, RH, and SB conducted all the interviews in this study. We ensured that none of the research assistants had prior relationship with the participants to avoid any undue influence.

The interviews were conducted face to face in a private setting, chosen by the study participants. Such settings included the participants’ office, clinic or reception rooms. The study goals and objectives were explained to the participants. All interviews were audiotaped. The interview as well as the completion of the sociodemographic questionnaire lasted for an average of 30 minutes.

### Ethical considerations

The study received ethical approval from the Institutional Review Board (IRB) at the American University of Beirut under protocol number SBS-2019-0059. Oral consent was received from all participants prior to participation, and each participant was assured of their anonymity and confidentiality. Study participants were handed a copy of the oral consent script, which included the contact information of the principal investigator as well as the IRB office (Additional file 2). The IRB approved this procedure for obtaining the oral consent as evidenced by the board’s stamp on oral consent script. This script served to document the consenting process. The IRB supported the oral in lieu of the written consent in order to protect the identity of the interviewees. Participants were informed that participation is voluntary and that they can decide to withdraw at any time during the interview.

### Data analysis

Socio-demographic characteristics were analyzed using IBM SPSS Statistics software. All in-depth interview discussions were transcribed verbatim, translated into English, and reviewed for accuracy. The analysis used a thematic approach consisting of 6 phases [32]. In phase 1, JA, and RH were immersed in the data by reading and re-reading each transcript to familiarize themselves with the information provided and create an initial framework for data coding. In phase 2, the data coding was initiated according to the framework, while keeping room for additional codes as they emerge. In phase 3, GHA, JA, ZJ, and RH kept a log of emerging themes, their definitions, and sample narrative illustrating each theme. In phase 4, the list of themes was refined through discussion among GHA, JA, ZJ, and RH and with FN. In phase 5, the final thematic framework was identified and linked to the final social phenomenon. In phase 6, a complete narrative of the findings was provided, whereby for each of the themes identified, the different views of the three study groups were compared. Similarities and differences were charted and contrasted in the perceptions of the participants with regards to factors that influence CAM use, using a triangulated approach. Findings were supported using quotes from interviewees for each themes and sub-theme.

### Increasing rigor

In terms of credibility, in-depth interviewers shared the same first language as participants thus facilitating communication with each other. Moreover, the interviewers verified and validated the discussion by confirming it with the participant during the interview. Transcripts were compared with the audio recordings to increase the accuracy of data interpretation. Data collection was stopped when saturation was reached. All conversations were audio recorded, transcribed verbatim, translated into English, and used as the main data repository. In terms of researcher credibility, trained interviewers conducted the interviews. In terms of reflexivity, the interviewers had no prior relationship with the participants. To avoid bias interpretation of the results, all research team members took part in the analysis where codes, themes, and subthemes were discussed. As for transferability, the triangulation enabled to gather perspectives from different population groups. The Consolidated criteria for Reporting Qualitative research (COREQ) Checklist [33] was followed when reporting this study (Additional file 3). In terms of dependability, the use of a topic guide in conducting the data collection provided consistency in repeating the data collection while observing variations among different participants.

## Results

### Sociodemographic characteristics

Table 1 describes the sociodemographic characteristics of participants from group 1 (Lebanese adults), group 2 (CAM providers), and group 3 (HCPs). The mean age of participants was 37.8, 47.8 and 36 years for groups 1, 2, and 3, respectively. Approximately, 71% of group 1, 46% of group 2, and 57% of group 3 were males. The majority of participants from group 1 (64.3%) and all subjects from groups 2 and 3 were employed.

**Table 1:** Sociodemographic characteristics of participants from groups 1, 2, and 3^a^

Participants’ characteristics	Group 1 (***n***=14)	Group 2 (***n***=13)	Group 3 (***n***=14)
Age (years)	37.8±17.7	47.8±12.9	36.0±12.8
Gender			
Female	4 (28.6)	7 (53.8)	6 (42.9)
Male	10 (71.4)	6 (46.2)	8 (57.1)
Education			
Up to primary school	0	0	0
Up to high school	6 (42.9)	2 (15.4)	0
University or higher (including Technical Diploma)	8 (57.1)	11 (84.6)	14 (100)
Employment Status			
Working	19 (64.3)	13 (100)	14 (100)
Not working	5 (35.7)	0	0
Marital Status			
Married	8 (57.1)	10 (76.9)	7 (50.0)
Single/Divorced/Widowed	6 (42.9)	3 (23.1)	7 (50.0)
Income			
600,001 – 1,999,000 ($401 – $1,332.9)	8 (57.1)	2 (15.4)	3 (21.4)
2,000,000 – 2,999,000 ($ 1,333 - $1,999.9)	1 (7.1)	3 (23.1)	1 (7.1)
≥ 3,000,000 (≥ $ 2,000)	3 (21.4)	2 (15.4)	6 (42.9)
Refused to answer	2 (14.3)	6 (46.2)	4 (28.6)

^a^Values in this table represent mean ±SD and n (%) for continuous and categorical variables, respectively

### General overview of interviews

The results of the thematic analysis of the interviews are presented within three main themes and their corresponding sub-themes: the understanding of CAM, the *Push* and the *Pull* factors. For each domain, a selection of participants’ quotes is included in this section. A more extensive list of the quotes for each domain are presented in Additional file 4.

### Understanding of CAM

Despite a few differences using the triangulation approach, the three study groups had similar perspectives on what CAM stands for and its various types.

The majority of participants from the three study groups understood CAM as an umbrella concept that encompasses all products and practices that are used to manage different health conditions other than CM. This was conveyed through statements like “*different than conventional medicine*”, “*not part of conventional medicine*”, and “*other than standardized medical care*”. Study participants also described CAM as everything that is “*cultural*”, “*traditional*”, and “*ancient*”.

Participants from all stakeholder groups had a generally good knowledge of the different types of CAM and all combined listed a total of 32 distinct practices belonging to the CAM categories. A number of listed practices were however not part of the conventional CAM categories such as exercise, drawing, psychological therapy, hypnosis, and a number of folkloric and cultural practices (e.g. use of natural soaps and salts), reflecting a certain degree of ambiguity in the conceptualization of CAM among participants. Herbal medicine was the most frequently listed type of CAM (58.5% of participants), followed by Chinese medicine (36.5%), and cupping therapy (Hujama) (26.8%). Around 22% of participants mentioned faith, religion, or a form of prayer as a type of CAM that people commonly resort to in Lebanon.

Participants from all groups clearly differentiated CAM from CM and made numerous comparisons between them. For Lebanese adults, the main difference between CAM and CM was that CAM is “*natural*”, “*non-chemical*”, and “*non-pharmacological*”, while CM is mostly referred to as chemical and invasive, having used terms like “*taking medications*”, “*injections*”, “*chemical pills*”, “*pharmacological*”, “*visiting the doctor*”, and “*surgical and physical medicine*” to refer to it.

A few adults described CAM as holistic health practices that engage the mind to promote health and physical wellbeing and focus on the interactions between the brain, body, and soul. In this group, a few participants distinguished between CAM and CM with regards to scientific legitimacy wherein CM was associated with scientific evidence and CAM was not. One Lebanese adult defined CAM as “*everything that is not pharmacological, has not been tested, and lacks supportive assessment*”.

The statements of the CAM providers generally echoed those of Lebanese adults as they viewed their therapies as natural and more “*holistic*”, “*multi-disciplinary*”, “*individualized*”, and “*empowering*” alternatives to CM. For CAM providers, the holistic nature of CAM was its primary distinguishing factor from CM, and the reason why they thought CAM was more effective than CM.

Examples of statements made by CAM providers include:

- G2_2: “*I believe that CAM is the way to heal everything because I believe that the mind is really powerful, and it controls every part of us*.”
- G2_3: “*CAM works on detoxifying the body, mind, and spirit and works on reestablishing these connections. To reach wellness you need a balance of the three”.*

CAM providers disagreed, both among themselves and with Lebanese adults, on what the scope of practice for CAM is and should be. While some acknowledged that CM treatments are necessary and indispensable when it comes to treating “complex” conditions such as cancer, and that CAM’s effectiveness is limited to rather less complex ailments, others believed that the practice of CAM should be better recognized given that CAM is the historical foundational basis of modern CM and “*the first medicine on earth*”. As asserted by one CAM provider:

- G2-9: *“Alternative medicine is not an accurate term; it [CAM] should be referred to as the main medicine. One hundred years ago, this type of medicine was available in all houses and its development resulted in today’s medicine that is being taught in universities”.*

As for Lebanese HCPs, the definition of CAM revolved primarily around the therapies that “*were not taught in medical school*” and that they “*prescribed to patients other than pills and medications*”. In general, HCPs expressed positive views towards CAM, describing it as a “*diverse field of medicine*” that is “*progressively growing*”. Some HCPs acknowledged that CAM might be a “*satisfying alternative*” to CM for patients as it may provide them with “*psychological relief*”, especially when traditional medicine “*cannot intervene*” or causes side effects. Only one HCP thought CAM was a field that is new and unknown in Lebanon and the Middle Eastern region in general.

### Push and Pull Factors

Participants from the three study groups expressed a range of views on the factors that drive CAM use in Lebanon, including the two a priori themes1) *Push* factors which drive Lebanese patients away from the use of CM; and 2) *Pull* factors which make CAM therapies more attractive treatment options. Within each of these themes, subthemes emerged from the data collection. We compared the perspectives of Lebanese adults with that of CAM providers and Lebanese HCPs with regards to these different subthemes.

### Push factors

- *Limitations of CM are gains for CAM*

Participants from all three study groups agreed among each other that failure of CM to treat conditions or alleviate symptoms is the primary factor that pushes patients away from CM and towards the use of CAM. Lebanese adults shared different experiences where they felt desperate after being told that they had incurable conditions, had developed resistance to biomedical treatments, or had experienced more suffering from conventional treatments, but had then found relief in CAM. The below quotes showcase different instances where CAM was used as a last resort out of desperation and loss of hope in CM:

- G1_1: *“I had a car accident that caused me neck pain which was extended to my arms. I visited many class (A) doctors in Lebanon who prescribed medications, cortisone, and physiotherapy, but nothing was beneficial. That is why I went for an osteopath. The first session was very painful, but after 3 days, the pain was reduced by 50% and after 15 days the feeling of numbness in my hands was completely gone*.”
- G1_4: *“I went to a doctor who did a lot of hearing tests and told me that my case has no cure and that I must live with it. Then my husband told my case to another doctor who prescribed this herbal medication. After taking it, I found it to be very beneficial.”*

CAM providers added that CM treatments are generally limited in the scope of care they provide, and that what patients often seek in CAM in terms of psychological support and individualized care is often not offered in conventional care. A few CAM providers said:

- G2_1: “*CAM is a holistic alternative that focuses on spiritual, psychological, biological, and other factors that are not addressed in the classical conventional medicine*”.
- G2_2: “*I also think in many cases the use of CAM is related to the limitations in medicine as medicine alone is not enough”*.

Participants from all study groups noted that patients often seek CAM therapies as a last resort after having tried conventional therapies. While only two Lebanese adults said they had dropped CM when it failed to bring them satisfaction, the majority combined CM with one or more CAM modality for the management of a given condition though to different extents. One HCP said:

- G3_3: “*People resort to CAM when they have tried traditional medicine and couldn’t get cured. It is very rare to see people start using CAM first for a given ailment before trying traditional medicine*”.

HCPs acknowledged that CAM could offer a viable treatment option to desperate patients when CM fails and shared their positive experiences on CAM and how it helped patients feel better. One HCP said:

- G3_1: “*I have seen cases such as vitiligo whereby years of traditional treatments didn’t cure the patient, then he used herbal medicine and got cured. Patient infertility is another example. The patient used CAM (herbal medicine) and then she got pregnant.”*
  b. *Distrust in the CM system*

Lebanese adults spoke of another major factor that pushes them away from CM and towards the use of CAM, which is their mistrust in Lebanese physicians’ knowledge and intentions when prescribing medication. Lebanese adults shared a common perception that HCPs are primarily driven by financial gain, and are influenced by pressure from pharmaceutical companies to prescribe their products. Lebanese adults and a few CAM providers believed that HCPs in Lebanon view the CAM industry as a threat to their business, which makes the intentions behind their advice on CAM questionable. One CAM provider said:

- G2_6: “*Traditional medicine practitioners might think that CAM providers are taking away their patients, so they start thinking in a selfish way, believing that CAM might decrease the number of patients, so they don’t encourage its use”.*

The below quote reflects Lebanese adults’ negative perceptions towards physicians’ knowledge and intentions.

- G1_1: *“Physicians have the knowledge [on CAM] but they don’t want to show patients that they trust CAM. They [physicians] think it [CAM] would substitute their work. They would say that they don’t believe in it and that it is not evidenced based.*
  iii. *Lack of a patient-centered approach in CM*

A combination of other factors related to specific aspects of the patient-physician interaction were also found to account for Lebanese adults’ dissatisfaction with CM. These included physician’s inadequate explanation of the reasons to advise against CAM, little time spent to discuss alternative treatment options with the patient, and physicians’ general lack of understanding of patients’ need for CAM, as shown in the excerpts below.

- G1_6: *“He [the physician] will not understand. He will tell me there are medications why would you take this herb”.*
- G2_1: “*Classical conventional medicine does not give enough time to the patient. Therefore, patients refer to CAM. The ideal timing and care that the patient usually seeks are found in alternative medicine*.”

HCPs attributed their negative attitude towards CAM to their lack of knowledge on its use and to their concerns over its safety, which are primarily related to the lack of experience and exposure to CAM in medical schools and the paucity of scientific studies on CAM. The below excerpts summarize HCPs point of view on this issue:

- G3_1: “*Health care providers aren’t knowledgeable about CAM. This is a weakness for me because we don’t study CAM in university because there aren’t enough studies on it. But it is being used very commonly. When we encounter someone using CAM we don’t know how to react, we don’t have the knowledge and we have to make a personal effort and search for information on it”.*
- G3_4: “*If a patient comes to me and tells me that they are taking a certain type of herbal medication, I will tell them that we don’t support it because we don’t have enough information on it. As a healthcare provider, I think that I don’t have much information about it because we have a kind of blockage towards it. We always stop their use and advise for drugs that are tested and that can be used safely without any side effects, unlike CAM whose side effects are not clearly known”.*

In addition to the mentioned *Push* factors, a number of CAM providers also believed that patients who start using CAM simply seek to “*avoid the chemicals*” and “*the side effects that are secondary to conventional treatments*” and use what they perceive as natural and safer alternatives.

### Pull factors

The three groups of the study population all addressed a number of “*Pull*” factors that make CAM an attractive treatment option, though their perspectives were, in some instances, divergent. These factors include the perceived effectiveness and safety of CAM, CAM therapies’ lower cost compared to CM treatments, as well as the significant role of social network in shaping their CAM knowledge and behaviors.

- *Perceived effectiveness and safety*

A few Lebanese adults believed that CAM therapies work on the biological aspect of the disease while others described its effects to be “*both biological and psychological*”. CAM providers believed CAM therapies to be effective in “*boosting immunity*”, “*reducing oxidation*”, “*increasing oxygen availability*”, and “*cleansing the body*”, which in turn would protect from diseases in early and preventative stages.

A few other CAM providers added that the holistic and non-chemical nature of CAM therapies evokes a certain degree of psychological relief and a sense of empowerment in patients, which adds another layer to its effectiveness.

When discussing the types and stages of diseases where CAM is effective the most, different opinions were shared. One Lebanese adult believed in CAM’s ability to “*decrease the progression of chronic diseases*”, while another stated that “*CAM is effective on acute diseases and conditions such as scars and inflammation*” only. On the other hand, only three HCPs believed in the effectiveness of CAM at all, except in cases where traditional medicine fails to treat such as in cases of infertility, vitiligo, and rheumatism.

While most Lebanese adults and CAM providers perceived CAM therapies to be natural and thus safe, participants from the HCPs group raised concerns over CAM therapies’ safety and elaborated on the reasons behind their reluctance to advise patients on CAM. HCPs emphasized the lack of scientific evidence behind the use of most CAM therapies, their unknown side effects and levels of toxicity, as well as the lack of regulation of the CAM industry in Lebanon. One HCP said:

- G3_1: “*Afterall, even though they [CAM] are herbs, they also contain chemicals and the reaction to them depends on the dosage. So as long as we don’t know the dosage or the type of CAM they [patients] are taking, patients are at great risk of developing side effects. Not to mention that some herbs are even poisonous*”.

b. *Cost*

Participants from all three study groups agreed that CAM’s lower cost compared to CM makes it a much more attractive option for patients who seek cheaper and cost-effective treatments to manage their ailments, especially when considering some CAM therapies such as prayer or laughter. Below are a few quotes to reflect the role of cost as an enabler of CAM use.

- G1_9: “*CAM also saves money. It is not necessary to take medications every time I have a sore throat. Each medication might cost around 20$, so I resort to herbal remedies first*”.
- G2_5: “*Laughter yoga is not costly, since all you need is to laugh and allow this laughter to happen”.*
- G3_7: *Patients prefer the easiest and the cheapest way, which is CAM, and then they seek medical advice if their case got complicated.*

iii. *Social support and culture*

According to most participants from all three study groups, a main motivator for the use of CAM in Lebanon is positive social influence. Individuals surrounding patients such as “*friends and family members play an essential role in influencing their use of CAM*”**,** and because “*people are affected by their surroundings, if individuals around them are using CAM and benefiting from it then they will automatically be encouraged to use it themselves*”. Additional file 4 summarizes the quotes that reflect the central role of social support in driving CAM use among the Lebanese people from the perspective of all three stakeholders.

A few Lebanese adults elaborated that they specifically rely on information from their network of people of older age and those living in rural areas, whom they thought had more experience and exposure to the Lebanese cultural practices. One Lebanese adult said:

- G1_9: “*I take advice from people of older generations such as my grandparents since during their time, traditional medicine was not that popular. I take advice especially from those who live in rural areas since they have a lot of experience with such herbs, and they know more than I do*”.

Moreover, Lebanese adults’ choice of therapy was found to be largely influenced by the common and time-honored practices that were passed on to them across generations through cultural heritage. A few HCPs believed that the Lebanese culture predisposes people to prefer all that is natural and non-medical.

Only a few participants from each group mentioned religious support as an important driver to the use of CAM in Lebanon, where “*some patients have religious ideologies and believe in CAM which motivates its use*”.

## Discussion

**Fig. 2:**
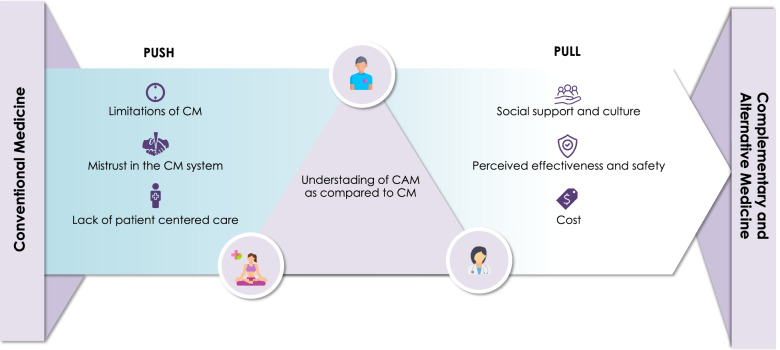
A triangulated approach to understanding CAM use: summary of findings

To the best of our knowledge, this is the first study to examine the factors that drive CAM use in Lebanon from the triangulated perspectives of Lebanese adults, CAM providers, and HCPs. By comparing and contrasting the perspectives of the different stakeholders, our analysis revealed important opportunities which, if addressed, may enhance the understanding of CAM and facilitate its integration within CM. The results of this study have been summarized in Fig. 2.

The study reveals that the three stakeholder groups exhibit a clear and coherent understanding of CAM, which may be stemming from the fact that the practice of CAM, particularly herbal medicine, is a rooted underpinning of tradition, culture, and religion in Lebanon, and a part of the collective memory of the people in Lebanon and the Arab region [16, 34]. Both historical and recent studies indicate that the Middle East region has long been distinguished with its rich repertoire of natural medicinal herbs [34]. Arab families still include in their medicinal inventory many of these herbs and practices which are commonly passed on across generations as part of family and cultural heritage, even though very few have had their medicinal properties investigated using evidence-based science [14].

Our findings show that Lebanese adults and CAM providers agree that dissatisfaction with CM and the general negative perception towards Lebanese physicians are two major factors that push the Lebanese people towards the use of CAM, which is in line with other findings from Lebanon and elsewhere [30]. Other push factors that were congruently mentioned by the different stakeholder groups in this study and supported by findings from other studies include the fear of CM therapies’ adverse effects, their high cost, and the belief that they may target one specific pathology rather than looking at the body from a holistic lens [35, 36].

Despite the dissatisfaction with many aspects of CM voiced in this study, CM was not generally condemned. Most Lebanese adults preferred using CAM as complementary treatments to CM and not as alternatives, even when they felt that conventional therapies did not meet their therapeutic goals. This attitude is likely to indicate that the Lebanese people do not trust CAM fully to replace CM, understand the limitations of both CAM and CM, and seek an integrated model of care where CAM therapies are accepted as complementary treatment options that fill in the gaps left by CM. This finding is encouraging as it constitutes a convergence point that all stakeholder groups agree with and could serve as the first building block in the discourse on CAM integration within CM.

While HCPs generally understood the advantages of CAM and acknowledged the shortcomings of CM that were indicated by the Lebanese adults and CAM providers, the majority expressed skepticism and reservation towards recommending CAM use. These were mainly due to the lack of scientific evidence, the concerns over CAM’s safety and effectiveness, and the absence of monitoring and regulation over the CAM field in Lebanon. In addition to that, most HCPs in this study doubted their level of knowledge on CAM as it is largely based on their own personal experiences rather than on academic/medical training. Due to these reasons, HCPs in this study felt more comfortable in dismissing any conversation on CAM, or in directing patients away from its use, often doing so without much elaboration, nor recognition of the impact of such dismissal on patient trust. One possible problematic outcome of such dismissal is that patients, who already anticipate this negative response, may choose not to discuss their concurrent use of treatments with their HCP, and as such may assume the entire burden of managing the interactions of CM and CAM without any medical guidance, which puts them at a great health risk [37, 38]. The considerable gap in CAM-related knowledge among HCPs has been previously reported in Lebanon and other Arab countries [35, 36, 39, 40], and is a well-cited reason for not recommending the CAM to patients worldwide [41–43]. Moreover, patients’ perception that HCPs lack interest and knowledge on CAM is a commonly cited reason for non-disclosure of CAM use, and limited education and training regarding CAM among HCPs has been shown to contribute to limited physician–patient dialogue regarding CAM [43]. Effective patient-physician communication is essential to establish a trusting and successful relationship and is known to greatly influence patients’ health outcomes [44].

In this study, the patient-physician trust was shown to be further weakened by a general negative perception of the Lebanese physicians as materialistic individuals who primarily seek to please pharmaceutical companies and achieve financial gains, a perception that was shared by both Lebanese adults and CAM providers. This negative perception of Lebanese physicians was also noted in a previous study [45]. Furthermore, as demonstrated in this study, a significant discrepancy exists between Lebanese adults - CAM providers and HCPs with regards to their views of CAM’s safety, which may further contribute to the non-disclosure of CAM use. While Lebanese adults consider CAM to be natural and thus safe and possibly not relevant in the biomedical treatment, most HCPs consider the CAM use to be scientifically unfounded and therefore better avoided, which means that the conversation on the CAM use may never be initiated by either party.

Another important underlining of this study is related to the relationship between HCPs and CAM providers, which was found to be built around rivalry and competition over shares in the health care market rather than on coordination and integration. The literature examining the relationship between CAM providers and HCPs is very limited but does suggest a lack of communication and coordination [46, 47]. Such a siloed model of health care not only signifies a fragmented care and a mutual exclusiveness of CAM and CM for the patient, but also reflects the struggle of the health care system to understand health and illness from the perspectives of all stakeholders involved.

### Implications and recommendations

A common denominator to most of the aforementioned concerns of the three study groups was the lack of knowledge and familiarity of the HCP with CAM, which was found to be hindering effective communication between the different parties involved, thus jeopardizing CAM’s safe and effective use. HCPs are naturally biased and sometimes incentivized to prescribe CMs just like CAM providers may be naturally biased to prescribe CAM therapies as the optimal and healthiest treatment option. Between the two biases, patients are the losers in the absence of proper guidance on CAM use and coordination between care providers.

Nevertheless, the findings of this study and other studies indicate that there is an impetus for change and an opportunity for interventions to increase the knowledge and familiarity of HCPs on CAM in Lebanon, which would in turn eliminate biases and improve the experience of patients with the conventional health care system. An 8-year follow-up survey conducted in a US-based academic medical center noted a remarkable rise in the percentage of physicians who are willing to recommend CAM in their practice from 44% to 77% following the introduction of monthly educational seminars, professional development courses, and readily accessible CAM targeted resources to medical students at the center, in addition to other interventions such as increasing research efforts on CAM and the addition of CAM services to the electronic ordering system of the center along with the other conventional services [48].

In light of the study’s findings, several recommendations can be put forward to ensure a safe and effective use of CAM products and services in Lebanon. These recommendations include the need to develop HCPs’ knowledge and communication skills so that they are able to communicate effectively and compassionately with patients seeking CAM therapies, without compromising trust, a value that is central to healthcare. The findings on HCPs’ acknowledgment of their lack of knowledge on CAM open a remarkable window of opportunity for the Ministry of Public Health (MoPH) to work collaboratively with the respective syndicates and professional societies to establish national programs for the continuous education of HCPs on CAM products, in collaboration with academic institutions. Other recommendations include the need to monitor and regulate CAM and CM use and provision in Lebanon as means to ensure patient safety. In order to maximize the impact of these recommendations, it is important to have a unified definition of CAM. To that end, it is recommended that the MoPH in collaboration with the main stakeholders, adopt a context specific definition of what constitutes CAM. In addition, more efforts should be put into research on CAM to provide evidence-based data on the effectiveness and safety of CAM in different health conditions. Specifically, these studies are to be conducted in association with conventional medical treatments, rather than being studied independently to replicate real-life scenarios and reflect patients’ CAM use behavior patterns. The findings of this study should be incorporated in both research and practice in order to promote a patient-centered approach to healthcare.

### Strengths and limitations

This is the first study to examine the factors influencing CAM use in Lebanon from the triangulated perspectives of Lebanese adults, CAM providers, and HCPs in Lebanon. Using a multi-stakeholder approach provides a more comprehensive and realistic view of the current CAM practice in Lebanon. Moreover, the qualitative nature of this study offers a unique and deeper understanding of the perspectives of the different stakeholders and the relationships between each other. However, the findings of this study are ought to be considered in light of a few limitations. A social desirability bias could not be ruled out, as participants may have formulated their answers in a way that they deem acceptable by the interviewer. However, the interviewers in this study were trained to maintain a neutral and non-judgmental attitude during data collection. The selection of interviewees may have not been representative of all the target groups, especially given that their selection did not account for geographic variability between those living in urban centers and those living in semi-urban/rural areas. The study did not account for the factors that may have deterred patients from using CAM, which could be also an important consideration in shaping consumption patterns. Future studies could possibly focus on this.

## Conclusion

In summary, challenges have been identified in bridging the views towards CAM between the patient-HCP-CAM provider nexus and to finding a common ground to support a broader perspective to health and wellness that avoids direct competition between CAM and CM. Although full alignment of perspectives of the different stakeholders is unrealistic, CAM discussions between stakeholders should remain open to enhance the physician-patient relationship and promote negotiation and informed empowerment rather than passivity and lack of trust.

## Supplementary Information

**Additional file 1.**

**Additional file 2.**

**Additional file 3.**

**Additional file 4.**

**Additional file 5.**

## Data Availability

The datasets generated and/or analyzed during the current study are found as supplementary material to this manuscript.

## Abbreviations

CAM: Complementary and Alternative Medicine
CM: Conventional Medicine
HCPs: Health care Providers
MENA: Middle Eastern and North African
COREQ: Consolidated Criteria for Reporting Qualitative Research
WHO: World Health Organization

## References

[1] National Center for Complementary and Integrative Health. Complementary, Alternative, or Integrative Health: What’s In a Name? 2016 [Available from: https://www.nccih.nih.gov/health/complementary-alternative-or-integrative-health-whats-in-a-name.

[2] National Center for Complementary and Integrative Health. Complementary and Alternative Medicine 2017 [Available from: https://nccih.nih.gov/news/camstats/2010/introduction.htm.

[3] World Health Organization. WHO Traditional Medicine Strategy 2002–2005 2002 [WHO/EDM/TRM/2002.1]. Available from: http://apps.who.int/medicinedocs/pdf/s2297e/s2297e.pdf.

[4] National Center for Complementary and Alternative Medicine. CAM Basics: What is Complementary and Alternative Medicine? 2011.

[5] Alzahrani AS, Price MJ, Greenfield SM, Paudyal V (2021). Global prevalence and types of complementary and alternative medicines use amongst adults with diabetes: systematic review and meta-analysis. Eur J Clin Pharmacol.

[6] Walker D-M, Tangkiatkumjai M. CAM use from western and asian perspectives: overview of different cultural beliefs of cam medicine and prevalence of use. Complement Altern Med Kidney Health. 2018:24–42.

[7] Tangkiatkumjai M, Boardman H, Walker D-M (2020). Potential factors that influence usage of complementary and alternative medicine worldwide: a systematic review. BMC Complement Med Ther.

[8] World Health Organization. Traditional medicine. WHO Fact Sheet No. 134 2008 [Available from: http://www.who.int/topics/traditional_medicine/en/.

[9] World Health Organization. Traditional and Complementary Medicine Policy. (MDS-3: Managing Access to Medicines and Health Technologies, Chapter 5) 2012 [Available from: http://apps.who.int/medicinedocs/en/m/abstract/Js19582en/.

[10] Naja F, Anouti B, Shatila H, Akel R, Haibe Y, Tfayli A. Prevalence and correlates of complementary and alternative medicine use among patients with lung cancer: a cross-sectional study in Beirut, Lebanon. Evid Based Complement Altern Med. 2017;2017. 10.1155/2017/8434697 PMC5587961 28912824

[11] Albabtain H, Alwhaibi M, Alburaikan K, Asiri Y (2018). Quality of life and complementary and alternative medicine use among women with breast cancer. Saudi Pharmaceut J.

[12] World Health Organization. WHO Traditional Medicine Strategy 2014-2023 2013 [ISBN 978 92 4 150609 0 (NLM classification: WB 55) ]. Available from: http://apps.who.int/medicinedocs/documents/s21201en/s21201en.pdf.

[13] GVR. Alternative And Complementary Medicine Market Analysis By Intervention (Botanicals, Acupuncture, Mind, Body, and Yoga, Magnetic Intervention), By Distribution Method, And Segment Forecasts, 2013 - 2025. Tech Rep. 2017.

[14] Naja F, Alameddine M, Itani L, Shoaib H, Hariri D, Talhouk S. The use of complementary and alternative medicine among lebanese adults: results from a national survey. Evid Based Complement Altern Med. 2015;2015. 10.1155/2015/682397 PMC4461758 26106436

[15] Naja F, Fadel RA, Alameddine M, Aridi Y, Zarif A, Hariri D (2015). Complementary and alternative medicine use and its association with quality of life among Lebanese breast cancer patients: a cross-sectional study. BMC Complement Altern Med.

[16] Naja F, Mousa D, Alameddine M, Shoaib H, Itani L, Mourad Y (2014). Prevalence and correlates of complementary and alternative medicine use among diabetic patients in Beirut, Lebanon: a cross-sectional study. BMC Complement Altern Med.

[17] Ghazeeri GS, Awwad JT, Alameddine M, Younes ZM, Naja F (2012). Prevalence and determinants of complementary and alternative medicine use among infertile patients in Lebanon: a cross sectional study. BMC Complement Altern Med.

[18] Farah N, Bilal A, Hibeh S, Reem A, Yolla H, Arafat T. Prevalence and correlates of Complementary and Alternative Medicine use among patients with lung cancer: a cross-sectional study in Beirut, Lebanon. Evid Based Complement Altern Med. 2017.

[19] Abou-Rizk J, Alameddine M, Naja F. Prevalence and characteristics of CAM use among people living with HIV and AIDS in Lebanon: Implications for patient care. Evid Based Complement Altern Med. 2016;2016. 10.1155/2016/5013132 PMC5168459 28050191

[20] Naja F, Alameddine M, Abboud M, Bustami D, Al HR (2011). Complementary and alternative medicine use among pediatric patients with leukemia: the case of Lebanon. Integrative Cancer Ther.

[21] Naja F, Fadel RA, Alameddine M, Aridi Y, Zarif A, Hariri D (2015). Complementary and alternative medicine use and its association with quality of life among Lebanese breast cancer patients: a cross-sectional study. BMC Complement Altern Med.

[22] Naja F, Mousa D, Alameddine M, Shoaib H, Itani L, Mourad Y (2014). Prevalence and correlates of complementary and alternative medicine use among diabetic patients in Beirut, Lebanon: a cross-sectional study. BMC Complement Altern Med.

[23] Alameddine M, Naja F, Abdel-Salam S, Maalouf S, Matta C (2011). Stakeholders' perspectives on the regulation and integration of complementary and alternative medicine products in Lebanon: a qualitative study. BMC Complement Altern Med.

[24] Broom A (2005). Using qualitative interviews in CAM research: a guide to study design, data collection and data analysis. Complement Ther Med.

[25] Franzel B, Schwiegershausen M, Heusser P, Berger B (2013). How to locate and appraise qualitative research in complementary and alternative medicine. BMC Complement Altern Med.

[26] Salamonsen A, Wiesener S (2019). “Then I went to a hospital abroad”: acknowledging implications of stakeholders’ differing risk understandings related to use of complementary and alternative medicine in European health care contexts. BMC Complement Altern Med.

[27] Welz AN, Emberger-Klein A, Menrad K (2018). Why people use herbal medicine: insights from a focus-group study in Germany. BMC Complement Altern Med.

[28] Sharp D, Lorenc A, Feder G, Little P, Hollinghurst S, Mercer S (2018). ‘Trying to put a square peg into a round hole’: a qualitative study of healthcare professionals’ views of integrating complementary medicine into primary care for musculoskeletal and mental health comorbidity. BMC Complement Altern Med.

[29] Sewitch MJ, Cepoiu M, Rigillo N, Sproule D (2008). A literature review of health care professional attitudes toward complementary and alternative medicine. Complement Health Pract Rev.

[30] Kharroubi S, Chehab RF, El-Baba C, Alameddine M, Naja F. Understanding CAM use in Lebanon: findings from a National Survey. Evid Based Complement Altern Med. 2018;2018. 10.1155/2018/4169159 PMC6083547 30147730

[31] Welz AN, Emberger-Klein A, Menrad K (2019). What motivates new, established and long-term users of herbal medicine: is there more than push and pull?. BMC Complement Altern Med.

[32] Braun V, Clarke V (2006). Using thematic analysis in psychology. Qual Res Psychol.

[33] Tong A, Sainsbury P, Craig J (2007). Consolidated criteria for reporting qualitative research (COREQ): a 32-item checklist for interviews and focus groups. Intern J Qual Health Care.

[34] Saad B, Azaizeh H, Said O (2005). Tradition and perspectives of Arab herbal medicine: a review. Evid Based Complement Altern Med.

[35] Samara AM, Barabra ER, Quzaih HN, SeH Z (2019). Use and acceptance of complementary and alternative medicine among medical students: a cross sectional study from Palestine. BMC Complement Altern Med.

[36] Hilal M, Hilal S (2017). Knowledge, attitude, and utilization of herbal medicines by physicians in the Kingdom of Bahrain: A cross-sectional study. J Assoc Arab Univ Basic Appl Sci.

[37] Lazar JS, O'Connor BB (1997). Talking with patients about their use of alternative therapies. Prim Care.

[38] Jou J, Johnson PJ (2016). Nondisclosure of complementary and alternative medicine use to primary care physicians: findings from the 2012 National Health Interview Survey. JAMA Int Med.

[39] Albadr BO, Alrukban M, Almajed J, Alotaibi K, Alangari A, Bawazir A (2018). Attitude of Saudi medical students towards complementary and alternative medicine. J Fam Commun Med.

[40] Hijazi MA, Shatila H, El-Lakany A, Ela MA, Kharroubi S, Alameddine M (2019). Beliefs, practices and knowledge of community pharmacists regarding complementary and alternative medicine: national cross-sectional study in Lebanon. BMJ Open..

[41] Al-Omari A, Al-Qudimat M, Hmaidan AA, Zaru L (2013). Perception and attitude of Jordanian physicians towards complementary and alternative medicine (CAM) use in oncology. Complement Ther Clin Pract.

[42] Roy V, Gupta M, Ghosh RK (2015). Perception, attitude and usage of complementary and alternative medicine among doctors and patients in a tertiary care hospital in India. Indian J Pharmacol.

[43] Patel SJ, Kemper KJ, Kitzmiller JP (2017). Physician perspectives on education, training, and implementation of complementary and alternative medicine. Adv Med Educ Pract.

[44] Matusitz J, Spear J (2014). Effective doctor–patient communication: an updated examination. Soc Work Public Health.

[45] Shaya B, Al Homsi N, Eid K, Haidar Z, Khalil A, Merheb K (2019). Factors associated with the public’s trust in physicians in the context of the Lebanese healthcare system: a qualitative study. BMC Health Serv Res.

[46] Penney LS, Ritenbaugh C, Elder C, Schneider J, Deyo RA, DeBar LL (2015). Primary care physicians, acupuncture and chiropractic clinicians, and chronic pain patients: a qualitative analysis of communication and care coordination patterns. BMC Complement Altern Med.

[47] Morin C, Desrosiers J, Gaboury I (2017). When, why, and how osteopaths and physicians communicate: lessons learned from the results of a mixed methods study. Intern J Osteopathic Med.

[48] Wahner-Roedler DL, Lee MC, Chon TY, Cha SS, Loehrer LL, Bauer BA (2014). Physicians' attitudes toward complementary and alternative medicine and their knowledge of specific therapies: 8-year follow-up at an academic medical center. Complement Ther Clin Pract.

