# Supplementary material for: A triangulated perspective for understanding CAM use in Lebanon: a qualitative study
Source: BMC Complement Med Ther. 2022 Aug 2;22:204. doi: 10.1186/s12906-022-03685-z (PMC9347103; doi:10.1186/s12906-022-03685-z)
Supplement: Supplementary file 1 — Additional file 1. [file 12906_2022_3685_MOESM1_ESM.docx]

| **Domain** | **Group A: Lebanese adults**  **(healthy, chronic diseases, users, non-users)** | | **Group B: CAM providers** | | **Group C: Health care providers** | |
| --- | --- | --- | --- | --- | --- | --- |
|  | **Question** | **Prompt** | **Question** | **Prompt** | **Question** | **Prompt** |
| **Understanding of CAM** | What is CAM? | Definition, types, examples, purpose, effects, side-effects | What is your understanding of CAM? | Definition, types, examples, purpose, effects, side-effects | What is your understanding of CAM? | Definition, types, examples, purpose, effects, side-effects |
|  |  |  | Have you received any academic training/education in CAM? Please elaborate. | N/A | Have you received any academic training/education in CAM? Please elaborate. | N/A |
| **Push Factors** | What pushes you away from CM towards the use of CAM? | Dissatisfaction with CM? Persistent pain? Looking for new treatment options?  Avoiding CM side effects?  Doctor-patient relationship? | What pushes patients/clients away from CM towards the use of CAM?  (Note: perspective of CAM providers about patients/clients). | Dissatisfaction with CM? Persistent pain? Looking for new treatment options?  Avoiding CM side effects?  Doctor-patient relationship? | What pushes patients/clients away from CM towards the use of CAM?  (Note: perspective of HCPs about patients/clients). | Dissatisfaction with CM? Persistent pain? Looking for new treatment options?  Avoiding CM side effects?  Doctor-patient relationship? |
|  | To what extent are HCPs knowledgeable about CAM in Lebanon? | N/A | To what extent are HCPs knowledgeable about CAM in Lebanon?  (Note: perspective of CAM providers about themselves). | N/A | To what extent are HCPs knowledgeable about CAM in Lebanon?  (Note: perspective of HCPs about themselves). | N/A |
| **Pull Factors** | Do you think CAM, in general, is safe? Do you think CAM is safe in Lebanon? | What makes you think it is safe/unsafe? Regulation? Non-chemical nature? | Do you think CAM, in general, is safe? Do you think CAM is safe in Lebanon?  (Note: perspective of CAM providers about patients/clients and themselves). | What makes you think it is safe/unsafe? Regulation? Non-chemical nature? | Do you think CAM, in general, is safe? Do you think CAM is safe in Lebanon?  (Note: perspective of HCPs about patients/clients and themselves). | What makes you think it is safe/unsafe? Regulation? Non-chemical nature? |
|  | Do you think CAM is effective? | In what type of diseases?  Share your experience. | Do you think CAM is effective?  (Note: perspective of CAM providers about patients/clients and themselves). | In what type of diseases?  Share your experience. | Do you think CAM is effective?  (Note: perspective of HCPs about patients/clients and themselves). | In what type of diseases?  Share your experience. |
|  | Do you think CAM is accessible in Lebanon? | What makes it accessible?  Cost?  Availability? | Do you think CAM is accessible in Lebanon?  (Note: perspective of CAM providers about patients/clients and themselves). | What makes it accessible?  Cost?  Availability? | Do you think CAM is accessible in Lebanon?  (Note: perspective of HCPs about patients/clients and themselves). | What makes it accessible?  Cost?  Availability? |
|  | Who is your primary source of information on CAM? | Media? Family/friends? HCP?  Do you think they are credible? | Who is your primary source of information on CAM?  (Note: perspective of CAM providers about patients/clients and themselves). | Media? Family/friends? HCP?  Do you think they are credible? | Who is your primary source of information on CAM?  (Note: perspective of HCPs about patients/clients and themselves). | Media? Family/friends? HCP?  Do you think they are credible? |
