# Supplementary material for: A triangulated perspective for understanding CAM use in Lebanon: a qualitative study
Source: BMC Complement Med Ther. 2022 Aug 2;22:204. doi: 10.1186/s12906-022-03685-z (PMC9347103; doi:10.1186/s12906-022-03685-z)
Supplement: Supplementary file 2 — Additional file 2. [file 12906_2022_3685_MOESM2_ESM.pdf]

Institutional Review Board
American University of Beirut

08 APR 2019

RECEIVED

Oral Consent Form

Protocol Title: Perceptions of Complementary and Alternative Medicine (CAM) among users, providers and health care workers in Lebanon: A qualitative study

IRB#:

Principal Investigator: Dr. Farah Naja

You are invited to participate in a research study that will include 10 to 15 participants from each of the following target populations: (a) Lebanese adults (users/non-users of CAM) aged 18-65 years, (b) CAM providers (wellness center managers, yoga instructors, laughter therapists, owners of CAM product outlets, herbalists, sheikhs, and priests); and (c) HCWs (physicians, nurses, dietitians, and pharmacists). The purpose of this study is to explore the perception of CAM among users/non-users, providers, and health care workers in Lebanon to obtain better understanding of the knowledge, attitudes and practices, as well as enablers and barriers of CAM use. To be eligible to participate, you must be either (1) Lebanese aged 18-65 years, or (2) one of the following CAM providers: wellness center manager, yoga instructor, laughter therapist, owner of CAM product outlets, herbalist, sheikh, priest, or (3) one of the following health care workers: physician, nurse, dietitian, pharmacist.

Recruitment

Lebanese adults aged 18-65 years will be recruited using flyers posted around the university and shared on AUB's Faculty of Agricultural and Food Sciences Facebook page. Subjects will include both CAM and non-CAM users. Subjects will include both CAM and non-CAM users, of different socio-economic statuses. Moreover, CAM providers and HCWs will be recruited from the catchment areas of the American University of Beirut Medical Center (AUBMC), as well as from the Makassed General Hospital (MGH). CAM providers -including wellness center managers, yoga instructors, laughter therapists, owners of CAM product outlets, and herbalists- will be invited to participate in the study via flyers posted at wellness centers and CAM outlets in the catchment areas of AUBMC and MGH. Furthermore, Dar Al Fatwa and Maronite Patriarchate will be contacted to nominate two sheikhs and two priests, respectively. The nominated sheikh and priest will be further invited and recruited by the research team using the invitation script. HCWs will be introduced to the study and invited to participate via a departmental meeting that will take place at each hospital -AUBMC and MGH. Pharmacists will be invited using flyers distributed at pharmacies within the catchment areas of AUBMC and MGH.

Interested participants will contact the research assistant who will explain the study as well as screen the potential participants for eligibility. If eligible, the participant and research assistant will decide on a convenient time and place to meet where the latter would feel comfortable and private to discuss his/her personal opinion. Before starting the interview, the interviewer will provide a brief description of the purpose of the study, the process of data collection, the risks and benefits as a result of their participation, and that they can withdraw from the study at any time without any consequences. Oral consent will be obtained from each participant, where a copy of the form will be kept with the participant.

Institutional Review Board
American University of Beirut

09 APR 2019

APPROVED

What will you do if you choose to participate in this study?

If you agree to participate, you will take part in a 30 minutes in-depth interview to explore your perception on the use of CAM therapy, including your knowledge, attitudes and practices, drivers and enablers, as well as barriers of CAM use. You will also be asked to complete a short demographic questionnaire. You will be asked permission to audio record the discussion. You have the right to refuse to participate in this study, to answer particular questions, or to audio record the discussion. The interview is completely anonymous and all the information that you will provide will remain confidential. Your individual privacy will be maintained in all published and written data resulting from the study as we will not be documenting your personal information. There are no direct benefits and no anticipated risks associated with this study. You will receive an in-kind incentive for the amount of $10 cash as a compensation for your time and contribution. The in-kind incentive is not contingent upon completing the entire study. You will receive the in-kind incentive during the research visit if you agree to participate in this study. Records will be monitored and may be audited by the IRB while assuring confidentiality.

This study will help us acknowledge and bring awareness of CAM use to the public and to health care professionals which could help promote the safety of CAM users. The goal is to provide more evidence for the community to make informed decisions about CAM. Please understand that your participation is voluntary and that you have the right to withdraw your consent or discontinue participation at any time without penalty. During the interview, you may skip answering any question just by saying "skip". You should keep this document with you. Do you have any questions about our study before we begin?

Statement by investigator

I have explained to the participant the study in detail including the proceedings and any disadvantages. I have answered all questions clearly to the best of my abilities.

Additional Information

If you have any questions, please contact Dr. Farah Naja on 01-350000 ext. 4504, or by email to fn14@aub.edu.lb. If you have further questions, you can contact the University's Review Board to discuss your rights on 01-350000 ext. 5445, or by email to irb@aub.edu.lb.

**Participation in the Study**
______**YES,** I would like to participate in this study.
______ **NO,** I do not like to participate in this study.

Institutional Review Board
American University of Beirut

09 APR 2019

APPROVED

**Permission to Audio Record the Discussion**
I would like to record the discussion so as to make sure that I remember accurately all the information you provide. I will keep these digital audio-tapes in a password-protected file saved on the investigator's computer in a folder, which is also password-protected. You may still participate in the interview if you do not want to be taped.<br><br>______**I PERMIT** audio recording the discussion.<br><br>______**I DO NOT ALLOW** audio recording the discussion.

**Permission to Quote from the Discussion**
When analyzing and reporting the data, we might need to use quotes from the discussion. We will ensure confidentiality by anonymizing transcripts and using codenames instead of your name.<br><br>______**I PERMIT** quoting from my discussion.<br><br>______**I DO NOT ALLOW** quoting from my discussion.

Institutional Review Board
American University of Beirut

09 APR 2019

APPROVED
