# Supplementary material for: A triangulated perspective for understanding CAM use in Lebanon: a qualitative study
Source: BMC Complement Med Ther. 2022 Aug 2;22:204. doi: 10.1186/s12906-022-03685-z (PMC9347103; doi:10.1186/s12906-022-03685-z)
Supplement: Supplementary file 4 — Additional file 4. [file 12906_2022_3685_MOESM4_ESM.docx]

**Appendix 2: Example of Interview Excerpts**

| **Main Study Themes** | **Quotes** |
| --- | --- |
| Understanding of CAM - Comparing CAM to CM | G1_3: “*What I know about CAM is that they are the medical products and practices that are  not part of the standardized medical care […].”*  G2_1: “*CAM is everything that is not included in the classical conventional medicine*”.  G3_1: “*Everything that we haven’t studied in medicine, is considered CAM to me such as acupuncture and herbal medicine*”. |
| Push factors - Limitations of CM | G1_8: “*Patients resort to CAM once they don’t find themselves responding to traditional medicine*”.  G1_2: “*Why would you refer to chemotherapy to cure cancer, which requires suffering and spending a lot of money, instead of doing it by faith*.”  G2_2: “*I also think in many cases the use of CAM is related to the limitations in medicine as medicine alone is not enough. In certain cases, people get disappointed and lose trust in conventional medicine and so automatically a lot of them rebel against the medical world and go towards CAM*.”  G3_2: “*Sometimes we see that the treatments we give do not work and that is when patients go towards alternative medicine which sometimes actually works*.” |
| Push factors - Distrust in the CM system | G1_10*: “Physicians are influenced and put under pressure of these [pharmaceutical] companies”.*  G2_1: “*HCP are against CAM because it is a competition. The greatest barrier to CAM use is the lack of support from physicians*”.  G2_16: “*Other doctors discourage the use of CAM. Physiotherapists react materialistically towards it and discourage people from using Hujama. Business should be discarded from medicine*”. |
| Push Factors - Lack of patient-centered care | G2_1: “*Classical conventional medicine does not give enough time to the patient. Therefore, patients refer to CAM. The ideal timing and care that the patient usually seeks are found in alternative medicine*.” |
| Pull Factors - Social support and culture | G1_10: *“Yes, I would get encouraged to try CAM following the recommendations of family members or friends”.*    G1_11: *“Of course, I trust the experiences of family members and friends. If they used a herb, I would also use it”.*  G2_2: “*People are affected by their surroundings, so if people around them are using CAM and benefiting then they themselves will automatically start using CAM”*.    G2_1: “*What pushes the patient onto using CAM are friends and family, etc. because it is discussed through them that it is a natural remedy that lacks any side effects*”.    G3_1: “*If someone heard that someone else with the same condition used a certain CAM therapy and was cured, they will use themselves*”. |
| Pull Factors - Perceived effectiveness and safety | G1_2: “*Yes, of course CAM is effective and efficient, out of personal experience. […] I believed in CAM [healing through faith] 100% when I experienced it myself*”.  G1_6: *“I think it [CAM] is effective on all types of diseases*”.  G2_16: “*Hujama, for instance, can never hurt you but can heal most diseases*”. |
| Pull Factors - Cost | G1_4: *“It [CAM] might help patients more in terms of cost savings since they would not need to do regular checkups with the physician. People spend a lot of money on physician checkups. Whereas with CAM, a person might only need 1 session at the physician”.*  G1_2: “*Healing through the church does not cost money”.*  G1_8: “*Also, in some cases, the traditional medicine is expensive […]”.*  G1_9: “*CAM also saves money. It is not necessary to take medications every time I have a sore throat. Each medication might cost around 20$, so I resort to the herbal remedies first*”.  G1_10*: “You might pay 200,000 Lebanese pounds for using medications and not benefit. But as for CAM its cost is less, more effective, and appropriate for the lower social class.”*  G2_5: “*Laughter yoga is not costly, since all you need is to laugh and allow this laughter to happen”.*  G2_3: “*Cost is an enabler because it is cheaper than conventional medicine*”.  G3_7: “*Patients prefer the easiest and the cheapest way, which is CAM, and then they seek medical advice if their case got complicated”.*  G3_7: “*Cost is an enabler to use CAM since CAM might be cheaper than traditional medicine*”. |
